# Altered intraperitoneal immune microenvironment in patients with peritoneal metastases from gastric cancer

**DOI:** 10.3389/fimmu.2022.969468

**Published:** 2022-09-02

**Authors:** Kazuya Takahashi, Kentaro Kurashina, Hironori Yamaguchi, Rihito Kanamaru, Hideyuki Ohzawa, Hideyo Miyato, Shin Saito, Yoshinori Hosoya, Alan Kawarai Lefor, Naohiro Sata, Joji Kitayama

**Affiliations:** ^1^ Department of Gastrointestinal Surgery, Jichi Medical University, Shimotsuke, Japan; ^2^ Department of Clinical Oncology, Jichi Medical University, Shimotsuke, Japan

**Keywords:** peritoneal metastasis, flowcytometry (FCM), peritoneal macrophage, gastric cancer, peritoneal immunity, peritoneal lymphocytes

## Abstract

**Background:**

The peritoneal cavity contains many site-specific immune cells which constitute a unique immune microenvironment. However, it is unclear how the local immune signature is altered in patients with peritoneal metastases (PM).

**Methods:**

Peritoneal lavage fluid or ascites were obtained from 122 patients with various stages of gastric cancer (GC). Cells recovered from peritoneal fluids were immunostained with mAbs for lymphocyte-, macrophage- and tumor cell-specific antigens and the frequencies of leukocyte subsets and antigen expression levels were evaluated with multi-color flowcytometry.

**Results:**

The proportions of CD8(+) T cells, CD3(+)CD56(+) NKT-like cells, and CD3(-)CD56(+) NK cells to CD45(+) leukocytes were significantly reduced in patients with PM compared to those without PM. In patients with PM, the rates of CD8 (+) T cells and NKT-like cells correlated inversely with the tumor leukocyte ratio (TLR), the relative frequency of CD326(+) tumor cells to CD45(+) leukocytes. In contrast, the proportion of CD19(+) B cells was significantly increased in patients with PM, and their proportion correlated positively with the TLR and peritoneal carcinomatosis index (PCI) score. In patients with PM, CD14(+) macrophages tended to be increased with enhanced expression of CD14, CD16 and a M2-macrophage marker, CD163. In particular, macrophages in patients with high TLR contained many granules with high side scatter and CD14 expression in their flow profile compared to those without PM.

**Conclusion:**

PM are accompanied by a drastic change in phenotypes of lymphocyte and macrophage in the peritoneal cavity, which might be involved in the development and progression of intraperitoneal tumor growth.

## Introduction

Gastric cancer (GC) is an important cause of cancer mortality and morbidity, being the fifth most frequently diagnosed cancer and the fourth leading cause of cancer death globally (1). Peritoneal metastases (PM) are common site of recurrence in patients with GC, especially for the scirrhous type with serosal exposure (2–4). PM are often associated with intestinal obstruction, hydronephrosis and/or accumulation of ascites, which significantly reduces the quality of life and is associated with an extremely poor prognosis (5–7). Elucidation of the molecular and cellular mechanisms responsible for tumor progression in the peritoneal cavity is extremely challenging, and necessary to develop novel strategies for the prevention and treatment of this dismal condition.

The development of PM is a multistep process, from the detachment of malignant cells from the serosal surface of the primary tumor, survival in the microenvironment of the abdominal cavity; attachment of free tumor cells to the peritoneal surface and invasion of the basement membrane; and tumor growth with the onset of angiogenesis (8, 9). Host immunity plays crucial roles in the process of metastasis formation (10, 11). Previous studies have shown that the peritoneal cavity usually contains many free-floating immune cells of various phenotypes which sustain peritoneal homeostasis and prevent local inflammation (12, 13). Since they can mediate direct contact with tumor cells exfoliated from the serosal surface of primary tumors, these immune cells in the peritoneal fluids are thought to play critical roles in the pathophysiology of PM.

The human peritoneal surface has markedly distinct immunological features in comparison to systemic immunity with abundant resident macrophages and B-1 cells, predominantly CD8(+) over CD4(+) T cells, as well as abundant soluble factors in the peritoneal fluid (14–17). However, little is known about how the immune microenvironment in the peritoneal cavity is modified in the process of the development of PM. In a previous study (18), we distinguished tumor cells and leukocytes in the peritoneal fluids of patients with GC using flowcytometry and calculated the relative frequency of CD326(+) tumor cells CD45(+) as the tumor leukocyte ratio (TLR). The results suggest that the TLR is an excellent biomarker to estimate peritoneal tumor burden and predict the outcome of the patients with PM. In this study, we used multicolor flowcytometry and performed a more detailed analysis of the phenotypes of intraperitoneal immune cells to estimate the changes in the immune microenvironment associated with PM.

## Materials and methods

### Patient and samples

Peritoneal lavage fluid or ascites was obtained from 122 patients with gastric cancer (GC) patients treated in the Department of Gastrointestinal Surgery, Jichi Medical University from April 2016 until January 2022. Among these patients, 75 underwent radical gastrectomy with curative intent and 47 patients with PM confirmed by staging laparoscopy received chemotherapy. For macrophage analyses, 31 patients with PM and 48 patients without PM were included. No patients received chemotherapy before surgery. From patients with substantial volumes of ascites, 10-20 ml of ascites was withdrawn. In patients without apparent ascites, the peritoneal cavity was irrigated with 500ml of normal saline and 250 ml of the fluid was recovered before surgical maneuver. In patients with PM, the peritoneal carcinomatosis index (PCI) score was determined after laparoscopic observation based on the original method (19). The final score was decided by discussion of two expert surgeons. Written informed consent was obtained from all patients. This research was conducted in accordance with the Declaration of Helsinki and was approved by the Ethics committee of Jichi Medical University (RIN21-HEN005)

### Monoclonal antibodies

Fluorescein Isothiocyanate (FITC)-conjugated mAb to CD4, phycoerythrin (PE)-conjugated mAb to CD16, CD56, allophycocyanin (APC)-conjugated mAb to CD14, CD8, BV605-conjugated mAb to CD11b, CD3, BV711-conjugated mAb to CD163, CD19 were purchased from BioLegend (San Diego, CA). FITC-conjugated mAb to CD66b was purchased from BIO-RAD (Hercules, CA). APC-conjugated mAb to CD326 (EpCAM) was purchased from Miltenyi Biotec (Auburn, CA). Fc-blocker, FVS780 were purchased from Becton-Dickinson (San Jose, CA) and DAPI from ThermoFisher Scientific (Waltham, MA).

### Flow cytometry

After centrifugation of ascites or peritoneal lavage fluid at 1500 rpm for 10min, pellets were resuspended and washed twice with PBS+0.02% EDTA. During this procedure, most of the cell clusters were dissociated to form single cell suspensions. The cells (1x10^5^~1x10^6^) were suspended in 100μl of PBS+0.02% EDTA and incubated with FVS780 for 15 min to label dead cells. After washing and Fc-blocking, cells were stained with conjugated mAbs for 30 min. After fixing and permeabilizing with Cytofix/Cytoperm (Becton-Dickinson, San Jose, CA), cells were stained with 0.1μg/ml DAPI and applied to BD LSR Fortessa™X-20 (Becton-Dickinson, San Jose, CA). For intracellular staining for IFN-γ, prepared cells (1 × 10^6^) were cultured in RPMI-1640 + 10% FCS for 4 hours at 37°C in the presence of 50 ng/ml PMA (Wako Chemical), 1 μg/ml ionomycin (Wako Chemical) and 5.0 μl/mL brefeldin A (BioLegend). The cells were harvested, fixed, permeabilized using fixation and permeabilization solution (BD Bioscience) according to the manufacturer’s instructions and stained with PE-conjugated IFN-γ or isotype control rat IgG1 with mAbs to CD3, CD8, CD56 and FVS780 to exclude dead cells. The ratio of IFN-γ positive cells was calculated in CD3(+)CD8(+), CD3 (-)CD56 (+) and CD3 (+)CD56 (+) gated areas using LSR FortessaTM X-20.

### Data analyses

Antigen expression and ratios of well-characterized immune cell populations were analyzed with Flow Jo™ software (Becton-Dickinson). The proportion of CD326(+) cell counts to CD45(+) cell counts was calculated and this value was defined as the tumor-leukocyte ratio (TLR). The flowcytometry data were also analyzed with dimensionality reduction and clustering using Cytobank software (Cytobank, Sanka Clara, CA). FCS file data gated on more than 15000 lymphocytes or CD14(+) macrophages were imported from 6 selected donors (3 patients with PM and 3 without PM) and *t-*SNE CUDA and FlowSOM were run with equal numbers of events per sample using Cytobank default parameters (iterations=750, perplexity=30, learning rate=200, early exaggeration=12).

### Statistical analysis

Statistical analyses were performed using Graph Pad Prism 8. Comparison between groups was evaluated by the Mann-Whitney U test. Correlation was analyzed using Spearman’s rank-order correlation. In all analyses, the standard for a significant difference was set at p < 0.05.

## Results

### The TLR in peritoneal fluids

**Figure 1:**
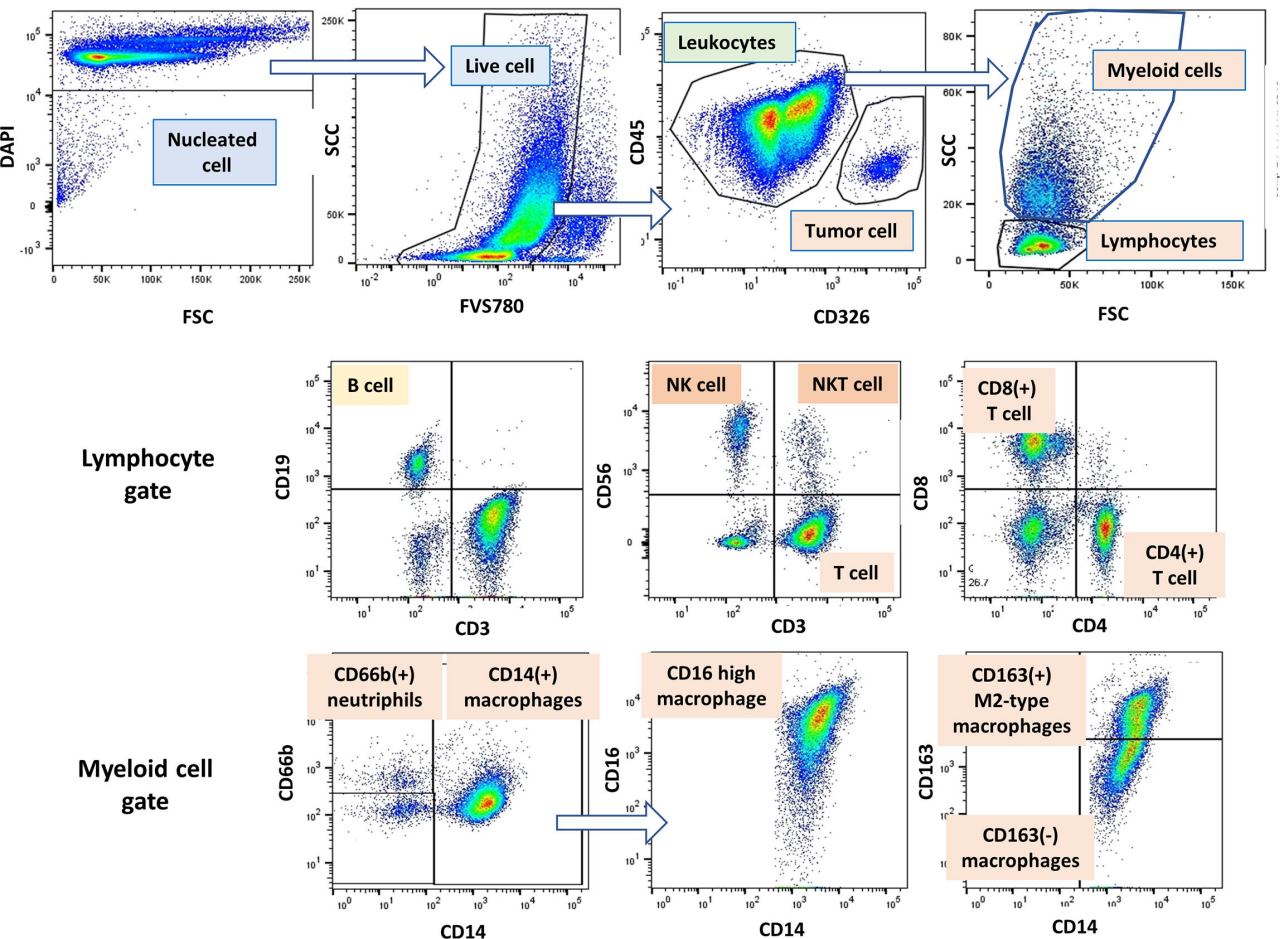
Gating strategy of lymphocytes, myeloid cells and tumor cells in peritoneal fluid. An example of gating method analyzed by Flow Jo software. Among the DAPI(+) peritoneal free cells, dead cells were excluded with FVS780. Tumor cells and leukocytes were defined as CD45(-)CD326 (+) and CD45(+)CD326(-) cells, respectively, and tumor leukocyte ratio (TLR) was calculated as described in Material and Methods. Among the leukocyte populations, lymphocytes and myeloid cells were distinguished as FSC/SCC profile, and expression of antigens was further examined in each cell population.

**Figure 2:**
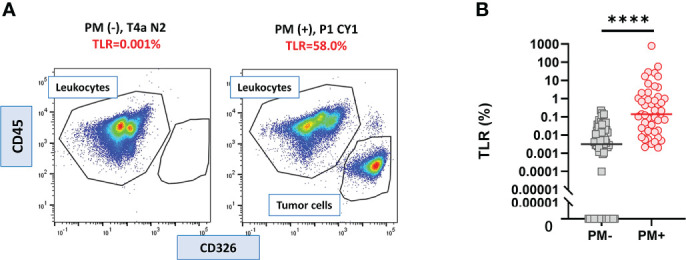
Tumor leukocyte ratio (TLR) in peritoneal fluids of the patients with or without PM. **(A)** Examples of flowcytometry profile of cells from the patients with or without peritoneal metastasis (PM). **(B)** TLR of 47 PM (+) and 75 PM (-) patients. Differences were evaluated using the Mann-Whitney *U* test. ****p <. 0001.

In all samples derived from peritoneal fluid, the expression of CD326 and CD45 was examined as epithelial and hematopoietic cell markers, respectively, and the ratio of CD326(+)CD45(-) tumor cells to CD326(-)CD45(+) leukocytes was calculated as the TLR (**Figure 1** upper panel). The value of the TLR had large variations from 0% to 790.3%. In 23/75 patients without PM, no CD326(+)CD45(-) tumor cells were counted among more than 10000 live cells whereas a small number of CD326(+) cells were detected in the other 52 patients (**Figure 2A** left panel), and thus their TLRs were calculated as 0-0.22% (median=0.0032%). In all 47 patients with PM, a significant number of tumor cells were detected in the flow profiles (**Figure 2A** right panel) and their TLRs were 0.002-790.3% (median=0.14%), which was significantly higher than in patients without PM (p<0.0001). (**Figure 2B**).

### The frequency of lymphocyte subsets in patients with and without PM

**Figure 3:**
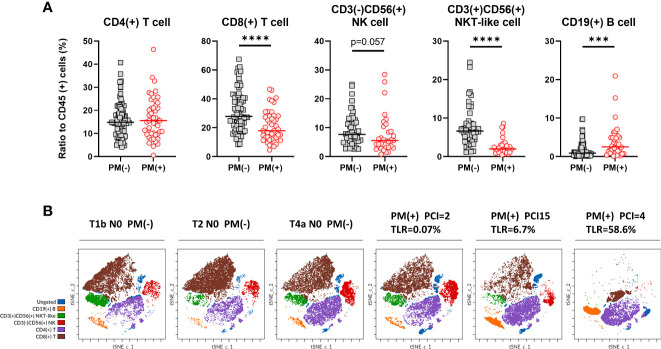
Lymphocyte subsets in peritoneal fluids of the patients with or without PM. **(A)** The proportion of CD4(+) or CD8(+) T cells, CD3(+)CD56(+) NKT-like cells, CD3(-)CD56(+) NK cells and CD19(+) B cells to CD45 (+) leukocytes were calculated in patients with or without peritoneal metastases (PM). Differences were evaluated using the Mann-Whitney *U* test. ****p <.0001, ***p <.001. **(B)** Clustering of lymphocyte subsets in representative patients. FCS data on lymphocytes of 3 patients with PM and 3 without PM were exported and dimensionality reduction and clustering were performed by t-SNE and FlowSOM using Cytobank software and coloring of the subsets determined by manual gating was shown.

As shown in **Figure 1** (middle panel), lymphocyte subsets were examined in lymphocyte gate and the results were shown in **Figure 3A**. The proportion of CD4(+) T cells to CD45 (+) leukocytes were not significantly different in patients with or without PM. However, the ratio of CD8 (+) T cells was significantly lower in patients with PM than in those without PM (median=17.8%, 4.6-46.7%, n=47, *vs* median=27.8%, 8.3-67.4%, n=75, p<.0001). The proportion of CD3(+)CD56(+) NKT-like cells was reduced in patients with PM (median=2.0%, 0.5-8.6%, n=31, *vs* median=6.6%, 1.1-24.5%, n=48, p<.0001). Similarly, the percentage of CD3(-)CD56(+) NK cells tended to be decreased in patients with PM (median=5.5%, 0.8-28.4%, n=31, *vs* median=7.6%, 2.5-25.0%, n=48, p=0.057). When simulated with PMA and ionomycin, CD8(+) T cells, CD3(+)CD56(+) NKT-like cells and CD3(-)CD56(+) NK cells produced high amounts of interferon (IFN)-γ which tended to be more than those in peripheral blood from healthy donor (**Supplementary Figure 1**).

In contrast, the ratio of CD19(+) B cells was significantly increased in patients with PM, although there were fewer than the number of T cells (median=2.5%, 0.1-20.9%, n=38, *vs* median=0.9%, 0.06-32.0%, n=64, p=0.0007). We also examined the phenotype analysis using dimensionality reduction and clustering method. As shown in **Figure 3B**, lymphocytes from 6 different patients with or without PM showed clearly different clustering and the frequencies of each subset showed similar trends with the results obtained with the traditional biaxial gating strategy.

**Figure 4:**
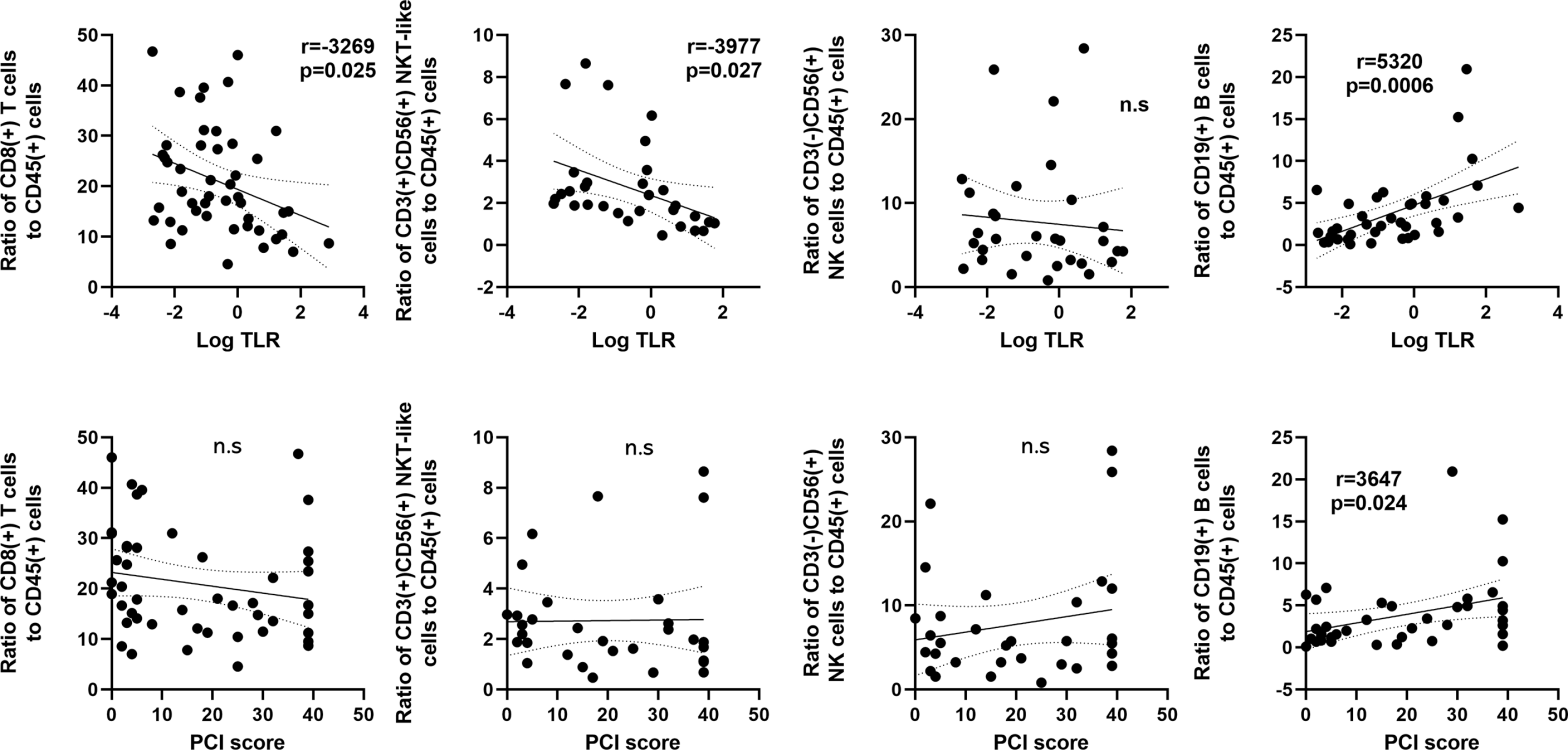
Correlation between frequencies of lymphocyte subsets and the tumor leukocyte ratio (TLR) or peritoneal carcinomatosis index (PCI) score in patients with peritoneal metastases. The X axis was plotted as log_10_ values of the TLR (upper) or PCI score (Lower). The Y axis was proportions of CD8(+) T cells, CD3(+)CD56(+) NKT-like cells, CD3(-)CD56(+) NK cells and CD19(+) B cells. Correlations were analyzed by Spearman’s rank-order correlation test. n.s., not significant.

The correlation between cell frequencies and tumor burden in the peritoneal cavity in patients with PM was then examined in patients with PM (**Figure 4**). The proportion of CD8(+) T cells correlated inversely with log_10_ values of the TLR (r=-0.3269, p=0.025). The TLR also showed a similar correlation with the ratio of CD3(+)CD56(+) NKT (r=-0.3977, p=0.027), but not with the ratio of CD3(-)CD56(+) NK cells. In contrast, the ratio of CD19(+) B cells correlated positively with the TLR (r=0.5320, p<0.001) and the peritoneal carcinomatosis index (PCI) score (r=0.3647, p=0.024). However, there was no association between the PCI score and the ratios of T, NK and NKT-like cells.

### Phenotypic changes of macrophages in patients with and without PM

**Figure 5:**
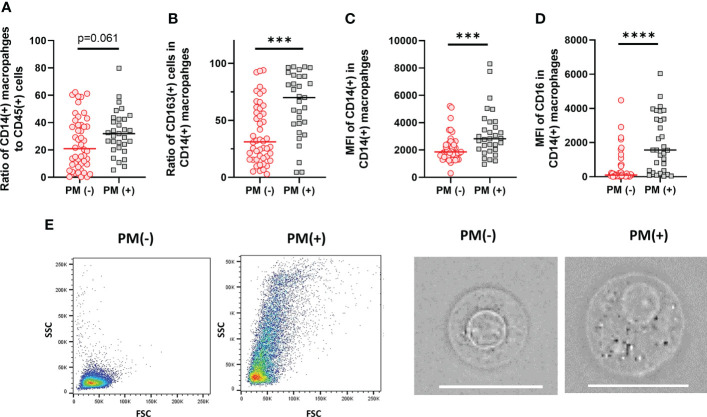
Characteristics of macrophages in the peritoneal fluids of the patients with or without peritoneal metastases (PM). **(A)** The proportion of CD14(+) macrophages to CD45 leukocytes. **(B)** The proportion of CD163(+) M2-type macrophages to CD14(+) total macrophages. Mean fluorescein intensities (MFI) of CD14 **(C)** and CD16 **(D)** in CD14(+) macrophages. **(E)** Examples of FSC/SCC profile and microscopic features of macrophages obtained from the patients with or without PM. Scale bar:20µm Differences were examined using Mann-Whitney *U* test. ***p <.001; ****p<.0001.

**Figure 6:**
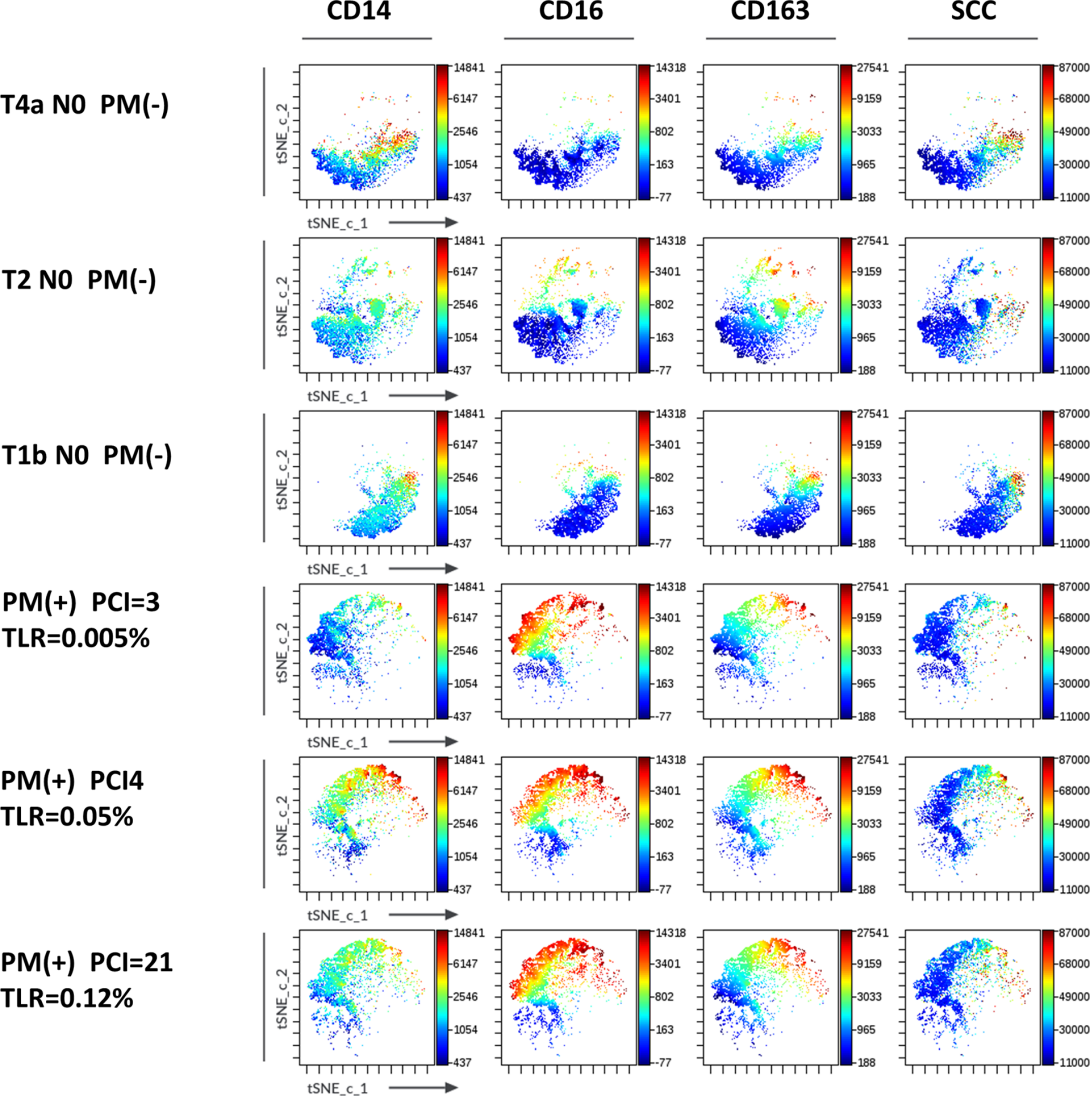
Expression of markers in *t-*SNE map of macrophages in the peritoneal fluids of the patients with or without peritoneal metastases (PM). FCS data on CD14(+) macrophages of 3 patients with (lower 3 panels) and without PM (upper 3 panels) PM were exported and *t-*SNE visualizations were produced using Cytobank software. Antigen expression levels for CD14, CD16, CD163 and SCC were overlayed as a color dimension on *t-*SNE maps.

Macrophage subsets were examined in 31 patients with PM and 48 patients without PM in myeloid cell gate (**Figure 1**, lower panel). The proportion of the total number of CD14(+) macrophages to CD45(+) leukocytes tended to be higher in patients with PM compared to those without PM, although the difference did not reach statistical significance (median=31.9%, 5.4-79.8%, *vs* median=20.9%, 0.3-62.2%, p=0.061) (**Figure 5A**). However, the expression of surface markers on the macrophages showed a marked difference in the presence of PM. The ratio of CD163(+) M2-type macrophages was significantly higher in patients with PM (median=70.1%, 4.4-97.1% *vs* median=31.5, 2.9-94.1, p=0.0001) (**Figure 5B**). The median of mean fluorescence intensity (MFI) of CD14 in patients with PM was 2817 (956-8304), which was significantly higher than that in patients without PM (median=1468, 308-5112, p=0.0008) (**Figure 5C**). The MFI of CD16 in the macrophages was increased more prominently in patients with PM compared to their counterparts (median=1564, 29-6036 *vs* median=100, 6-4477, p<0.0001) (**Figure 5D**). The macrophages in patients with PM, especially with high TLR cases showed high side scatter (SCC) in flow profile and contained large number of granules under microscopic observation as compared to those without PM (**Figure 5E**). In dimensional reduction plots with *t-*SNE analysis, CD14(+) monocyte populations showed clearly different distribution between patients with or without PM (**Figure 6**). As shown in upper 3 panels, cells with high CD14 and CD16 expressions were scarce in patients without PM. In contrast, macrophages with CD14^high^CD16^high^ phenotype markedly increased in lower 3 patients with PM. In addition, CD163(+) macrophages were mostly included in CD14^high^CD16^high^ cell populations and showed high SCC.

**Figure 7:**
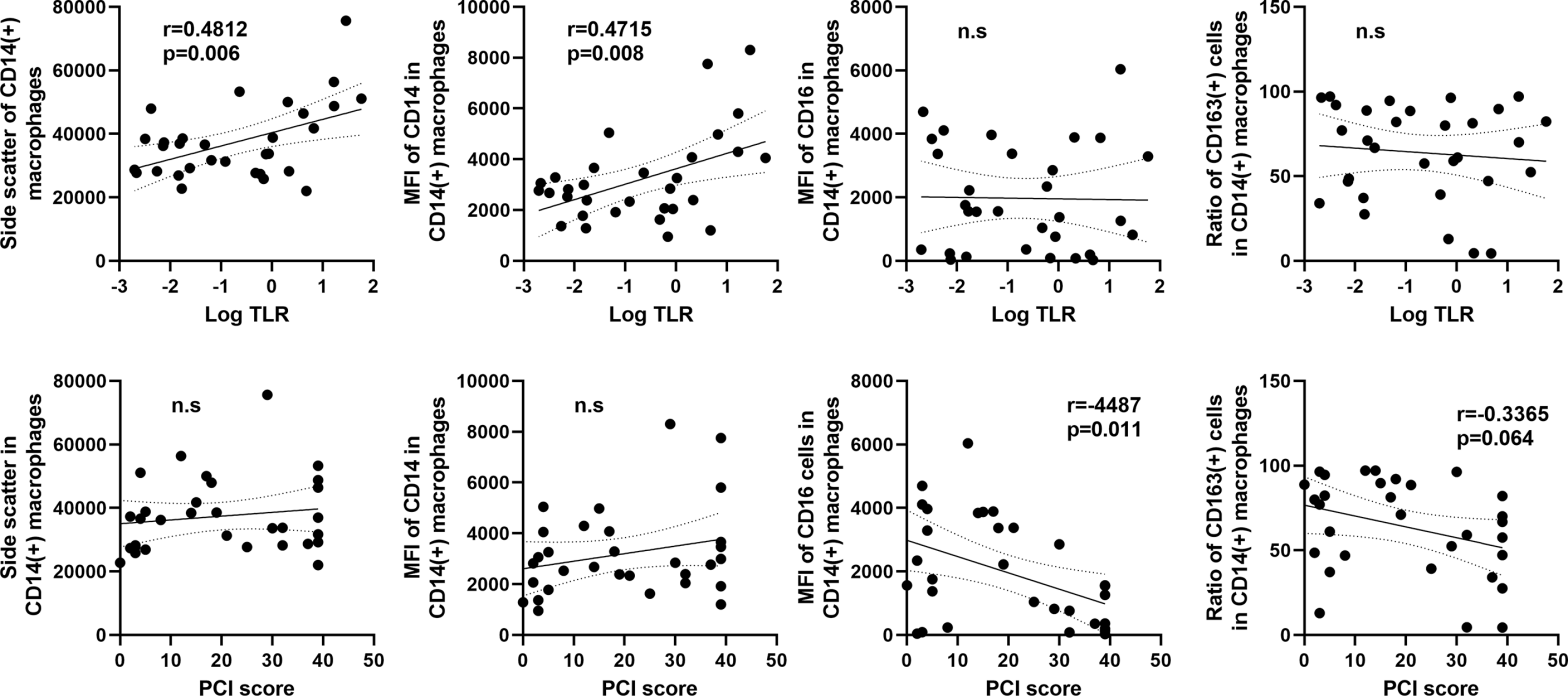
Correlation between macrophage features and the tumor leukocyte ratio (TLR) or peritoneal carcinomatosis index (PCI) score in patients with peritoneal metastases. The X axis was plotted aslog_10_ values of the TLR (upper) or PCI score (lower). The Y axis was the mean values of side scatter, mean fluorescein intensities (MFI) of CD14, CD16, and ratio of CD163(+) M2-type macrophages to CD14(+) total macrophages. Correlations were analyzed by Spearman’s rank-order correlation test. n.s., not significant.

As shown in **Figure 7**, the SCC of CD14(+) macrophages in flow profiles correlated positively with log_10_TLR (r=0.4812, p=0.006). The the MFI of CD14 also showed positive correlation with TLR. In contrast, the MFI of CD16 and ratio of CD163(+) M2-type macrophages did not show an association with the TLR but correlated inversely with the PCI score (**Figure 7**).

### Impact of the frequency of immune cell types on patient outcome

Finally, we examined the correlation of the frequency of these cell types and patient outcome. In patients without PM, overall survival (OS) and disease-free survival (DFS) tended to be better in patients with high ratio of CD8(+) T cell or CD3(-) CD56(+) NK cells compared to those with low ratio of these cells, although the difference did not reach the statistical significance (**Supplementary Figure 2**). On the other hand, OS and DFS of the patients with low ratio of CD14(+) macrophages tended to be better than their counterparts.

In patients who received intraperitoneal chemotherapy, peritoneal cytology (CY) was measured in 31 patients before treatment. The ratio of NK cells tended to be high in 13 CY (-) compared to 18 CY(+) patients (p=0.075) (**Supplementary Figure 3A**). In patients of the latter group, CY turned to be negative in 9, while remained positive in 5 patients after one course of chemotherapy. The proportion of CD3(-)CD56(+) NK cells was significantly higher in patients with negative CY change compared to counterparts (p=0.004)(**Supplementary Figure 3B**). Ascites was detected with CT image in 37 patients before the treatment and the change of ascitic volume was evaluated in 26 patients after 3 courses of chemotherapy. In those patients, ascites was greatly reduced or disappeared in 18 patients while increased or unchanged in 8 patients. The ratio of NK cells was significantly higher in 18 patients who showed good response as compared with other 8 patients (p=0.041) (**Supplementary Figure 3C**).

However, the ratios of these immune cells including NK cell showed no significant correlation with OS or progression free survival (PFS) of the patients with PM, presumably because the outcome of these patients is strongly dependent on the sensitivity of tumor cells to intraperitoneal chemotherapy.

## Discussion

The peritoneal cavity is the largest space in the human body and is lined by peritoneum consisting of a single layer of mesothelial cells and a sub-mesothelial basement membrane (20). In physiological conditions, the cavity contains a small amount of fluid containing various immune cells which are considered to patrol the peritoneal surface to preserve tissue homeostasis and secure tissue repair (12, 21). Although these immune cells are supposed to have a protective role against the growth of peritoneal tumors, the modifications of peritoneal immunity in the process of PM is unclear.

In the present study, the phenotypes of lymphocytes and macrophages in peritoneal fluids were found to be markedly altered by the presence of PM in patients with GC. First, the ratios of CD8 (+) T cells, CD3(+)CD56(+) NKT cells and CD3(-)CD56(+) NK cells, all of which have direct cytotoxic effects against tumor cells, were significantly decreased in patients with PM. Papadopoulos et al. reported that CD8(+) T cells and CD56(+) NK cells were depleted in the ascites from patients with PM of ovarian cancer (22). Yunusova et al. showed that NK cells in malignant ascites were decreased compared to those in ascites with benign ovarian tumors (23). The results in this study on gastric cancer are consistent with the previous reports regarding ovarian cancer.

In patients without PM, the ratios of CD8(+) T cells and CD3(-)CD56(+) NK cells in peritoneal fluids inversely correlated with the survival. Moreover, T, NK and NKT-like cells in peritoneal fluids produced high levels of intracellular IFN-γ as compared with circulating cells in healthy donor. This indicates that these peritoneal cells are not exhausted, but fully functional and suggest that a reduced number of these effector cells in the peritoneal cavity may be critical for the development of PM. In patients with PM, the proportions of CD8(+) T cells and NKT-like cells correlate inversely with the TLR, suggesting that the impaired effector functions may facilitate the rapid growth of free tumor cells in the peritoneal cavity. However, the frequency of these cells does not correlate with the PCI score. Since the PCI score reflects the macroscopic tumor burden in the peritoneum, free effector cells in peritoneal space may not be directedly related to the growth of established peritoneal tumors.

Instead, the proportion of B cells correlate positively with both TLR and PCI score. B cells can exert anti-tumor immunity through secretion of tumor-specific antibodies, promoting T cell responses, cytokine production, and a high density of tumor infiltrating B cells is generally associated with a better prognosis in patients with most cancers (24, 25). However, further evidence suggests that B cells can develop into regulatory B cells (B-regs) which produce high amounts of immunosuppressive cytokines leading to tumor progression (26, 27). From the results of this study, B cells in the peritoneal cavity are supposed to mostly share the functionality of B-regs to support the growth of PM.

Myeloid-derived suppressor cell (MDSC) is another important component of immune cells in cancer patients. In CD11b(+) myeloid cells, cells with CD15(+) CD14(-)HLA-DR(-) and CD14(+) CD15(-) HLA-DR low/(-) phenotype are considered as PMN-MDSC and M-MDSC in human peripheral blood, respectively (28). In this study, we separated CD11b(+) cells in peritoneal fluid into CD66b(+)CD14(-) polymorphonuclear cells (PMN) and CD66b(-)CD14(+) macrophages. As shown in **Supplementary Figure 4**, most of the CD66b(+)CD14(-) PMN were CD15(+) and HLA-DR(-) and inhibited the proliferation of T cells stimulated with coated anti-CD3 mAb (**Supplementary Figure 4**). On the other hand, CD66b(-)CD14(+) macrophages expressed high levels of HLA-DR and potently inhibited T cell proliferation. Although their phenotype was clearly different from M-MDSC in in peripheral blood, the macrophages might be considered as the M-MDSC in peritoneal cavity.

Indeed, the number of CD14(+) macrophages tended to be increased in patients with PM. More impressively, their phenotypes were drastically changed with enhanced surface expression of CD14, CD16 and CD163. Blood monocytes have been reported to be classified into two major types of monocytes, CD14^++^ CD16^−^ classical and the CD14^+^ CD16^+^ nonclassical monocytes (29, 30). A recent study showed that peritoneal macrophages are also classified into 3 phenotypes, CD14^++^CD16^-^, CD14^++^CD16^+^ and CD14^high^CD16^high^ group, although their expression pattern was different from blood monocytes (31). In that report, CD14^high^CD16^high^ macrophages highly expressed GATA6 as well as activation/maturation markers such as CD206 and HLA-DR and were considered to be a mature phenotype of human resident peritoneal macrophages. Although the expression patterns of CD14 and CD16 in macrophages in the present study are mostly consistent with the previous results, the expression levels of CD16 and CD14 were markedly increased in macrophages from the patients with PM. In particular, macrophages obtained from the patients with a high TLR showed extremely high expression levels of CD14 with high granularity. Previous studies showed that short-term incubation with lipopolysaccharide (LPS) decreased mCD14, whereas long-term treatment with LPS resulted in increased membranous CD14 expression on monocytes (32) as well as alveolar macrophages (33). From these data, the increased numbers of macrophages with the CD14^high^CD16^high^ phenotype in patients with PM are thought to be the resident macrophages chronically stimulated by tumor cells in the peritoneal cavity.

The CD14^high^CD16^high^ macrophages in patients with PM also highly expressed CD163, another marker of M2-type macrophages. In solid tumors, tumor associated macrophages (TAM) have been reported to be largely M2 phenotype macrophages, promoting a pro-angiogenic and immunosuppressive signal (34, 35). Previous studies have shown that macrophages co-cultured with gastric cancer cells likely differentiate into M2-type TAMs and have evident immunosuppressive effects on diffuse-type gastric cancer (36, 37). Moreover, peritoneal macrophages are shown to be phenotypically and functionally polarized to the M2 phenotype in patients with PM from gastric (38, 39) and ovarian (40) cancers. A recent study has demonstrated that cavity-resident macrophages with high expression of Tim-4 reduces the numbers of CD8+ T cells in pleural effusions and peritoneal ascites from patients with cancer (41). The results of the present study are consistent with those results and suggest that tumor cells exposed to peritoneal cavity can increase number of CD14^high^CD16^high^ macrophages which may play a pivotal role on the development of PM in patients with GC.

In conclusion, the number of T NK and NKT-like cells decreased while B cells and M2-type resident macrophages increased in the peritoneal cavity of the patients with PM. This altered immune cell composition can induce local immunosuppression which may be crucial for the growth of tumor cells exfoliated from the primary tumor. Further analysis focused on the function of these intraperitoneal immune cells, including single cell RNA sequence analyses, is necessary to define the immune landscape in peritoneal cavity which is related with the development and progression of PM. The results would provide useful information to identify novel clinical biomarkers and therapeutic targets for GC with possible peritoneal involvement.

## Data availability statement

The original contributions presented in the study are included in the article/**Supplementary Material**. Further inquiries can be directed to the corresponding author.

## Ethics statement

This study was reviewed and approved by Ethics Committee of Jichi Medical University. The patients/participants provided their written informed consent to participate in this study. Approval no: RIN21-HEN005.

## Author contributions

KT, KK, HY and JK designed and performed the experiments and analyzed the data. RK, HO, HM, SS, and YH contributed clinical samples, analysis tools or data. KT, AKL, NS, and JK drafted the manuscript. Critical revisions of the manuscript were made by all authors. JK approved the final version of the manuscript on behalf of all the authors. All authors contributed to the article and approved the submitted version.

## Funding

This work was supported by a Grant-in-Aid for Scientific Research from Japan Society for the Promotion of Science (20K22960, 21K16432) and Grand from Mochida Memorial Foundation for Medical and Pharmaceutical Research.

## Acknowledgments

We thank Ms Y. Hayakawa, J. Shinohara, H. Hatakeyama, and I. Nieda for technical and clerical works.

## Conflict of interest

The authors declare that the research was conducted in the absence of any commercial or financial relationships that could be construed as a potential conflict of interest.

## Publisher’s note

All claims expressed in this article are solely those of the authors and do not necessarily represent those of their affiliated organizations, or those of the publisher, the editors and the reviewers. Any product that may be evaluated in this article, or claim that may be made by its manufacturer, is not guaranteed or endorsed by the publisher.

## Supplementary material

Supplementary Figure 1 Cells from peripheral blood and peritoneal fluids were cultured in the presence of 50 ng/ml PMA and 1 μg/ml ionomycin with 5.0 μl/mL brefeldin A for 4 hours. The cells were immunestained with mAbs to IFN-γ as well as CD3, CD8 and CD56 and the ratio of cells positive for intracellular IFN-γ in CD3(+)CD8(+) T cell, CD3(-)CD56(+) NK cell, and CD3(+)CD56(+) NKT-like cell were evaluated with flow cytometry. **(A)** Flowcytometry profile of representative cases. **(B)** The rates of IFN-γ (+) cells in lymphocyte subsets derived from peritoneal fluids of 3 patients and circulating blood from 3 healthy donors.

Supplementary Figure 2 The patients without peritoneal metastasis were divided into two groups with the mean values of the ratios of CD8(+) T cells or CD3(-)CD56(+) NK cells and CD14(+) macrophages to CD45(+) leukocytes as the cut off, and their overall survival (OS) and disease-free survival (DFS) were examined with Kaplan-Meir method. P values were calculated with log-rank test.

Supplementary Figure 3
**(A)**The proportion of CD3(-)CD56(+) NK cells in positive (CY1) and negative (CY0) peritoneal cytology in patients with peritoneal metastasis. **(B)** The proportion of NK cells in patients with or without negative change of cytology after one course of intraperitoneal chemotherapy. **(C)** The proportion of NK cells in 18 patients with ascites. Ascites was evaluated in CT image before and after 3 courses of chemotherapy. Differences were examined using Mann-Whitney U test. *p <.05.

Supplementary Figure 4
**(A)**Flowcytometric profile of CD11b(+) myeloid cells in peritoneal fluid of 2 representative patients. **(B)** T cell proliferation assessed with standard CFSE dilution methods. Anti-CD3 mAb were incubated in 96 well plates at a concentration of 5μg/ml overnight. CD66b(+) PMN or CD14(+) macrophages were purified from the peritoneal fluid of a patient with positive selection using MACS method. PBMC (2x10^5^) labeled with CFSE were seeded in the plate with or without the purified the myeloid cells (5x10^4^). After 4 days culture, cells were harvested and stained with PE-conjugated anti-CD4mAb and APC-conjugated anti-CD8(+) mAbs and CFSE signal was analyzed by flow cytometry on gated either CD4(+) or CD8(+) T lymphocytes.
