# Supplementary figures and images for: Altered intraperitoneal immune microenvironment in patients with peritoneal metastases from gastric cancer
Source: Front Immunol. 2022 Sep 2;13:969468. doi: 10.3389/fimmu.2022.969468 (PMC9478385; doi:10.3389/fimmu.2022.969468)

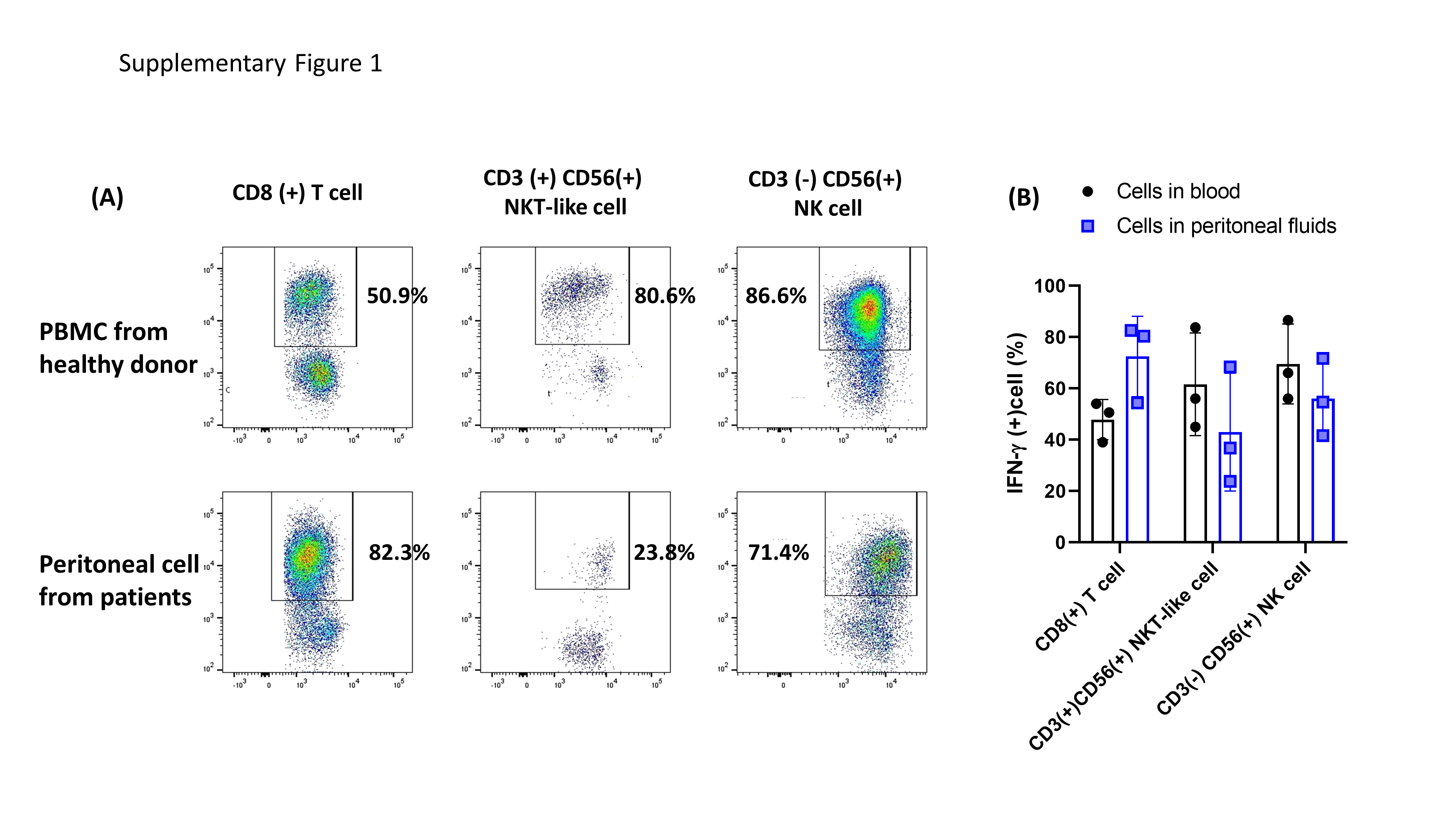

Supplement: Supplementary Figure 1 — Cells from peripheral blood and peritoneal fluids were cultured in the presence of 50 ng/ml PMA and 1 μg/ml ionomycin with 5.0 μl/mL brefeldin A for 4 hours. The cells were immunestained with mAbs to IFN-γ as well as CD3, CD8 and CD56 and the ratio of cells positive for intracellular IFN-γ in CD3(+)CD8(+) T cell, CD3(-)CD56(+) NK cell, and CD3(+)CD56(+) NKT-like cell were evaluated with flow cytometry. (A) Flowcytometry profile of representative cases. (B) The rates of IFN-γ (+) cells in lymphocyte subsets derived from peritoneal fluids of 3 patients and circulating blood from 3 healthy donors. [file Image_1.tif]

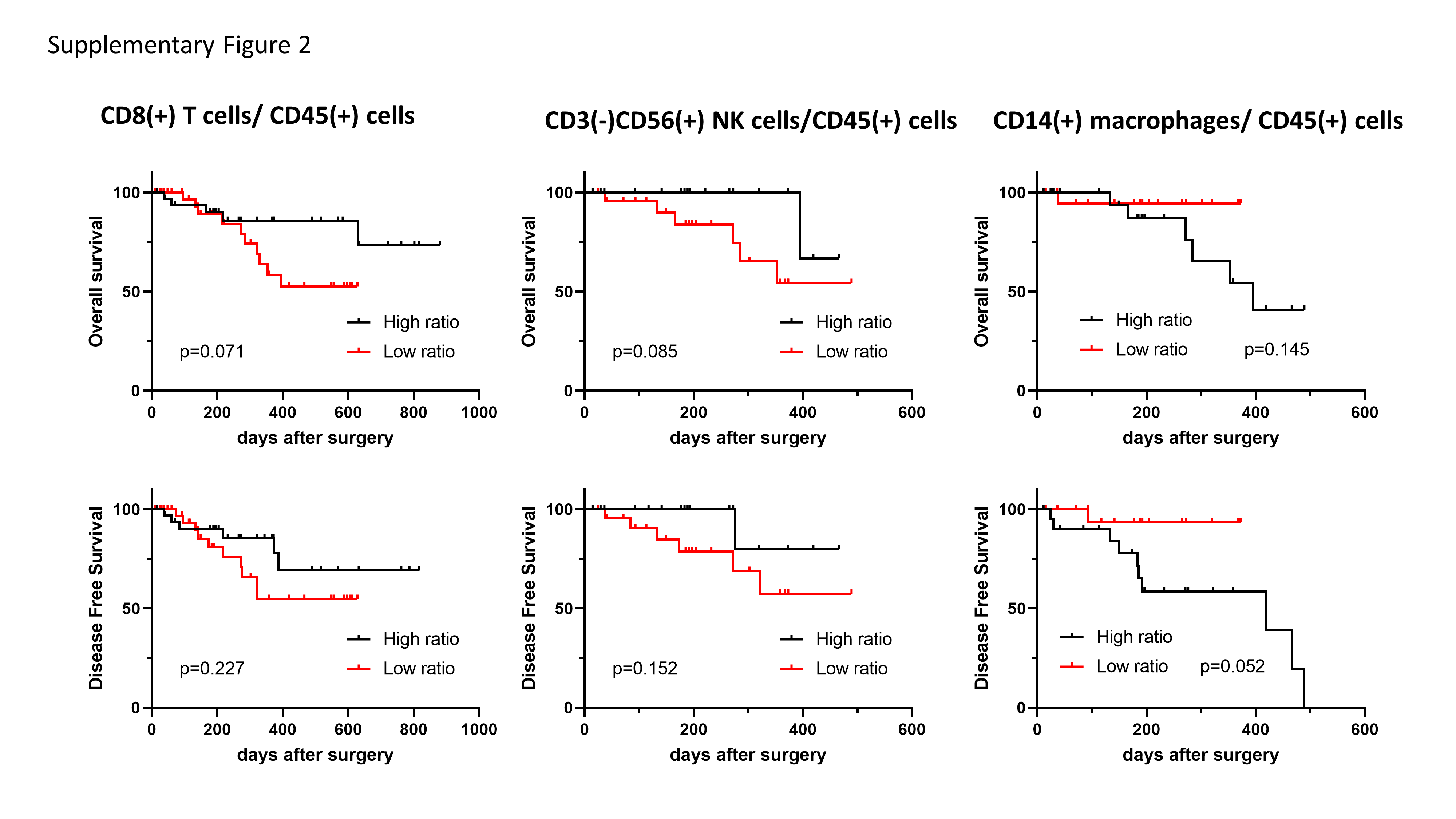

Supplement: Supplementary Figure 2 — The patients without peritoneal metastasis were divided into two groups with the mean values of the ratios of CD8(+) T cells or CD3(-)CD56(+) NK cells and CD14(+) macrophages to CD45(+) leukocytes as the cut off, and their overall survival (OS) and disease-free survival (DFS) were examined with Kaplan-Meir method. P values were calculated with log-rank test. [file Image_2.tif]

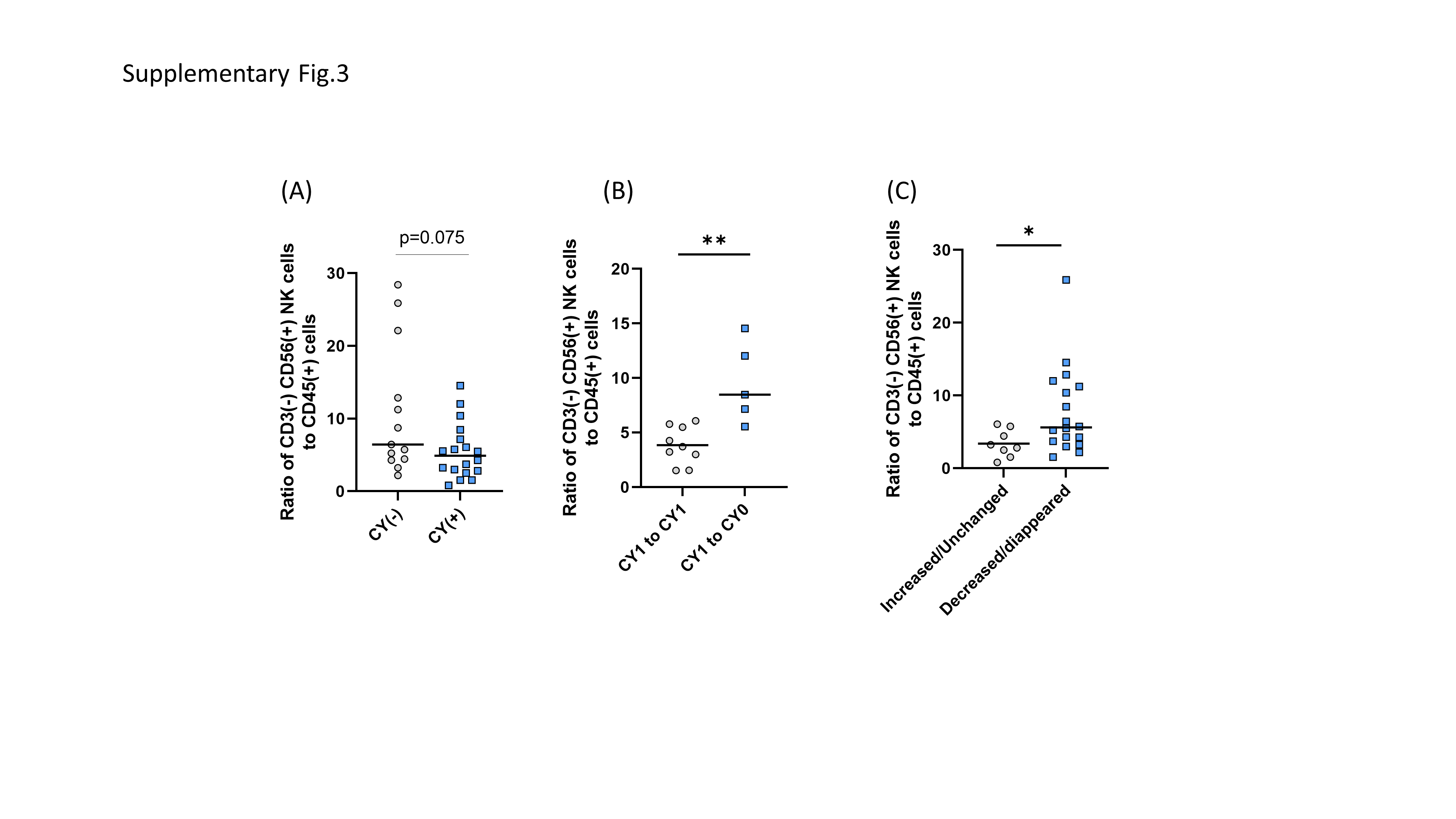

Supplement: Supplementary Figure 3 — (A)The proportion of CD3(-)CD56(+) NK cells in positive (CY1) and negative (CY0) peritoneal cytology in patients with peritoneal metastasis. (B) The proportion of NK cells in patients with or without negative change of cytology after one course of intraperitoneal chemotherapy. (C) The proportion of NK cells in 18 patients with ascites. Ascites was evaluated in CT image before and after 3 courses of chemotherapy. Differences were examined using Mann-Whitney U test. *p <.05. [file Image_3.tif]

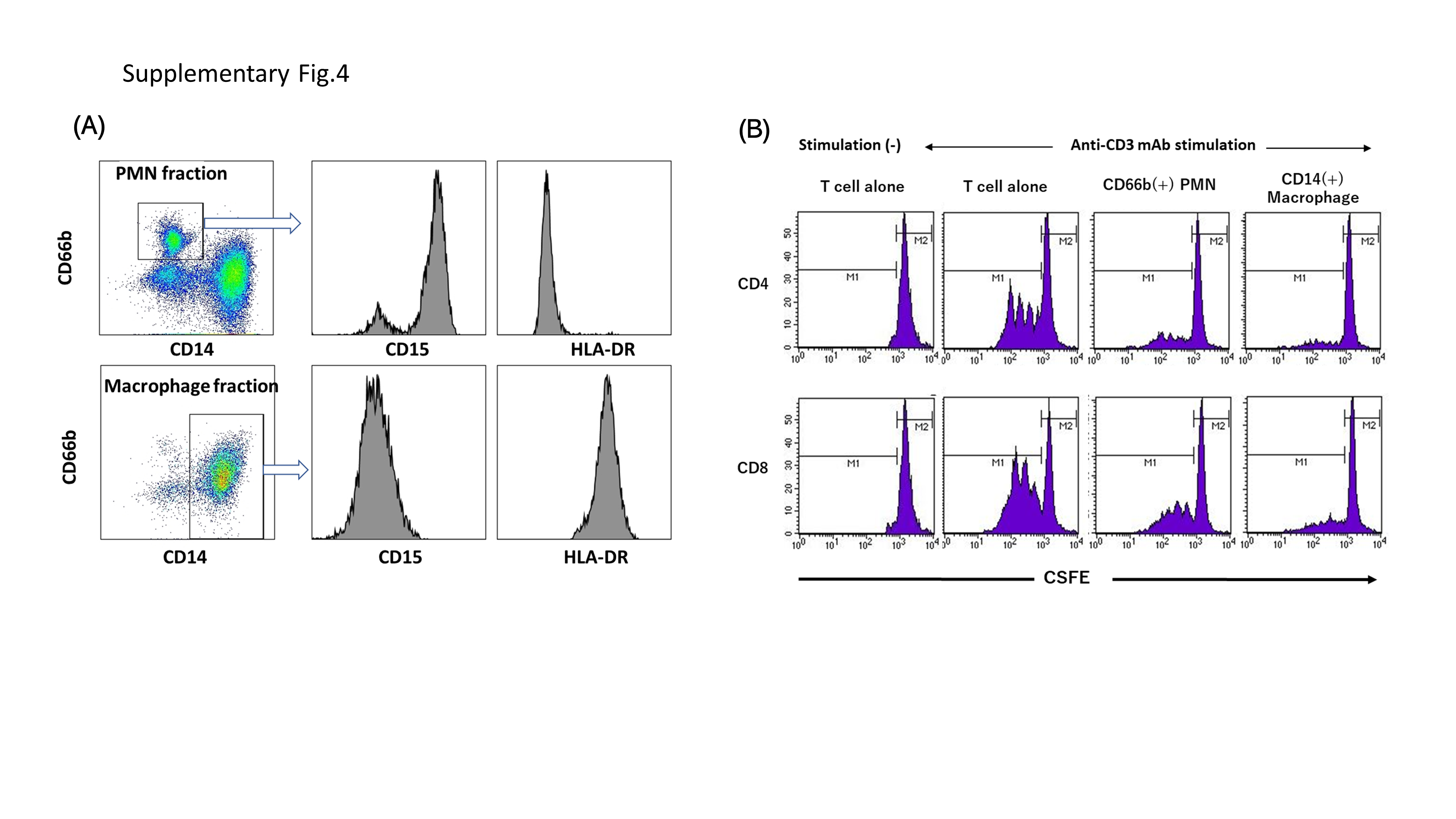

Supplement: Supplementary Figure 4 — (A)Flowcytometric profile of CD11b(+) myeloid cells in peritoneal fluid of 2 representative patients. (B) T cell proliferation assessed with standard CFSE dilution methods. Anti-CD3 mAb were incubated in 96 well plates at a concentration of 5μg/ml overnight. CD66b(+) PMN or CD14(+) macrophages were purified from the peritoneal fluid of a patient with positive selection using MACS method. PBMC (2x10^5^) labeled with CFSE were seeded in the plate with or without the purified the myeloid cells (5x10^4^). After 4 days culture, cells were harvested and stained with PE-conjugated anti-CD4mAb and APC-conjugated anti-CD8(+) mAbs and CFSE signal was analyzed by flow cytometry on gated either CD4(+) or CD8(+) T lymphocytes. [file Image_4.tif]
